# Assessment of serum biotin levels and its association with blood glucose in gestational diabetes mellitus

**DOI:** 10.1016/j.eurox.2023.100181

**Published:** 2023-02-17

**Authors:** N. Muthuraman, Reeta Vijayselvi, Yesudas Sudhakar P, Pamela Christudoss, Premila Abraham

**Affiliations:** a Department of Biochemistry, Christian Medical College, Vellore, Tamil Nadu, India; b Department of Obstetrics and Gynaecology, Christian Medical College, Vellore, Tamil Nadu, India; c Department of Clinical Biochemistry, Christian Medical College, Vellore, Tamil Nadu, India

**Keywords:** Diabetes, Gestational, Biotin, Insulin

## Abstract

**Aim:**

The incidence of gestational diabetes mellitus is increasing worldwide. Biotin is shown to improve glycemic status in diabetes mellitus. We wanted to study whether there is a difference in biotin levels between mothers with and without gestational diabetes mellitus (GDM), association of biotin with blood glucose, and with the outcome of GDM.

**Methods:**

We recruited 27 pregnant mothers with GDM and 27 pregnant mothers without GDM. We measured the biotin levels using enzyme linked immunosorbent assay (ELISA). We measured the blood glucose during OGTT and fasting insulin levels in the study participants.

**Results:**

We found that biotin levels were slightly decreased in mothers with GDM [271 (250,335)] as compared to control mothers [309 (261,419)], though it was not statistically significant (p = 0.14). Blood glucose levels were found to be significantly higher in GDM mothers as compared to control mothers during fasting, 1 h and 2 h plasma sample obtained during OGTT. Biotin was not significantly associated with blood glucose in pregnant mothers. Logistic regression analysis showed that biotin (OR = 0.99, 95 % CI = 0.99–1.00) has no association with the outcome of GDM.

**Conclusion:**

Ours is the first study to compare the biotin levels in GDM mothers and control mothers. We found that the biotin levels were not significantly altered in GDM mothers as compared to control mothers and biotin levels have no association with the outcome of GDM.

## Highlights

• This is the first study to compare biotin levels in GDM mothers with control mothers.
• Biotin levels were not significantly different in GDM mothers as compared to control mothers.
• Biotin has no association with the outcome of GDM.

## Introduction

Gestational diabetes mellitus (GDM) is defined by American Diabetes Association (ADA) as “diabetes diagnosed in the second or third trimester of pregnancy that was not clearly overt diabetes prior to gestation” [1]. The global prevalence of gestational diabetes mellitus is estimated to vary from 1 % to 30 % in different parts of the world [2]. Some of the known risk factors that can predispose the development of GDM in a pregnant mother are increased maternal age, obesity/overweight, family history of diabetes mellitus and cigarette smoking [3]. Insulin resistance and β-cell dysfunction are considered to be the main pathophysiology behind the development of GDM [4].

GDM can affect the outcomes of pregnancy by increasing the maternal and foetal complications. Short term complications of hyperglycaemia in GDM can lead to preeclampsia and increased propensity to undergo cesarean section in mothers. In infants born to mothers with GDM, complications such as macrosomia, shoulder dystocia, neonatal hypoglycemia and increased requirement for admission to intensive care units are commonly seen [5]. Meta-analysis results clearly shows that women who have GDM during pregnancy are at an increased risk of developing type 2 diabetes mellitus, as compared to the women who were normoglycemic during pregnancy [6]. Particularly women who were diagnosed to have GDM using International Association of Diabetes and Pregnancy Study Group (IADPSG) criteria are at higher risk of developing prediabetes and type 2 diabetes mellitus in the future [7].

Biotin is a water-soluble vitamin which acts as a coenzyme for carboxylase enzymes that is involved in the metabolism of carbohydrates and lipids [8]. Biotin is proposed as one of the natural supplements that can improve insulin sensitivity and glucose uptake in skeletal muscle [9]. Biotin is shown to have its hypoglycemic effect by increasing the expression of hepatic glucokinase gene and thereby effectively decreasing the blood glucose levels [10]. Trials using high dose biotin for type 1 diabetics have found promising results such as good glycemic control and better response to insulin insensitivity [11]. Also, there are animal studies which have shown that administration of high dose biotin can improve glucose tolerance in rats [12], [13]. Our pilot study showed that mega doses of biotin supplementation in diabetic rats lead to better pregnancy outcomes [14]. This prompted us to think whether biotin supplementation would be beneficial to GDM mothers in terms of reducing maternal and fetal complications. To pursue that, we thought that it would be relevant and informative to compare the biotin levels in mothers with and without GDM. We hypothesised that the biotin levels can be low in mothers with GDM as compared to the normoglycemic mothers and can be associated with the outcome of GDM. In this study we assessed the biotin levels in the pregnant mothers and looked for its association with blood glucose, insulin, and the outcome of GDM.

## Methods

### Study participants

This study was approved by the Institutional review board of Christian Medical College, Vellore. Pregnant woman in the gestational age of 24–28 weeks, who were referred for an oral glucose tolerance test (OGTT) from antenatal clinic in Obstetrics and Gynaecology – unit 4 of CMC Vellore, were recruited for the study after obtaining informed consent. The required sample size to show the difference in biotin levels between women with GDM and those without GDM was found to be 24 in each group, with 90 % power and alpha error of 5 %. Calculations were based on publication by Donald M. Mock et al. [15]. Only those who gave informed consent to participate in the study were included. Those who were unwilling to participate, known diabetic, on biotin supplements and with any pregnancy related complications were excluded from the study. Based on the one step (75 g of oral glucose) OGTT results, as per The International Association of the Diabetes and Pregnancy Study Groups (IADPSG) criteria (fasting blood glucose ≥92 mg/dL, or 1 h blood glucose ≥180 mg/dL, or 2 h blood glucose ≥153 mg/dL) they were grouped as pregnant women with GDM and pregnant women without GDM (control).

## Biotin estimation

Biotin concentration was estimated in the fasting blood sample that was collected at the time of OGTT. We used IDK® Biotin ELISA K 8141 kit to estimate the biotin concentration in the serum sample. We followed the instructions given in the manual with inclusion of appropriate standards and control samples to carry out the assay.

## Blood glucose estimation

Glucose levels were estimated in blood sample collected during fasting, 1 h and 2 h during OGTT. Glucose levels were estimated by glucose oxidase peroxidase method using Roche Cobas 8000c system in Clinical biochemistry department with strict adherence to quality control.

## Insulin levels

Fasting blood sample collected at 24–28 weeks of gestation were used to measure insulin levels. Insulin levels were measured using Siemens CLIA, following the instructions laid out in the kit with strict adherence to quality control. Homeostasis model assessment of insulin resistance (HOMA-IR), was calculated using fasting insulin levels and fasting blood glucose level by appropriate formula as described earlier[16].

## Statistical analysis

Statistical analysis was done using R version 4.2.1. RStudio: Integrated Development Environment for R. RStudio, PBC, Boston, MA URL http://www.rstudio.com/. Normality of the data was tested using Shapiro-Wilk test. Wilcoxon Rank Sum test (Mann Whitney U test) was done to compare the median between two groups. Correlational analysis between the variables were done using Spearman correlation test. Simple logistic regression analysis was done to test the association of biotin with GDM outcome.

## Results

### Baseline characteristics of the study participants

**Fig. 1:**
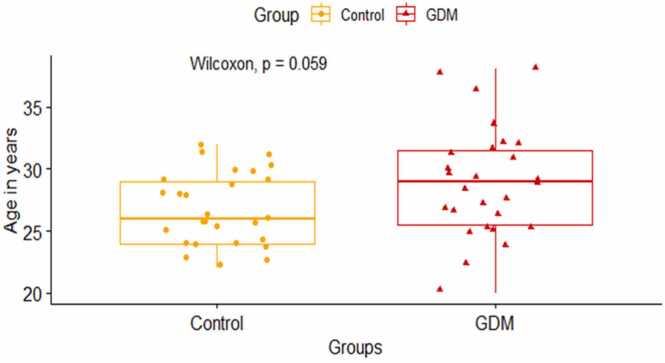
Comparison of median (IQR) age between GDM mothers and control using Wilcoxon Rank Sum test (Mann Whitney *U* test).

The baseline characteristics of the study participants are given in Table 1. The median age of pregnant mothers with GDM is slightly higher than the mothers without GDM and it approached close to statistical significance (p = 0.059, Fig. 1). As has been reported before the risk of gestational diabetes mellitus increases as the age during which the mother gets pregnant increases, which corroborates with our study’s finding.[17].

**Table 1:** Baseline characteristics of the study participants.

Variable	N	Control, N = 27^*1*^	GDM, N = 27^*1*^	
Age in years	54	26[24,29]	29[26,32]	
Fasting blood glucose (mg/dL)	54	80 [76,86]	89 [84,95]	
1 h blood glucose during OGTT (mg/dL)	54	135 (126, 152)	178 (152, 188)	
2 h blood glucose during OGTT (mg/dL)	54	109 (106, 116)	144 (124, 158)	
Biotin (ng/L)	54	309 (261, 419)	271 (250, 335)	
Fasting Insulin (μU/mL)	54	8.3 (5.5, 11.1)	10.0 (5.1, 13.2)	
HOMA-IR	54	1.70 (1.05, 2.25)	2.10 (1.15, 3.15)	
^*1*^ Median (IQR)

## Blood glucose concentration

**Fig. 2:**
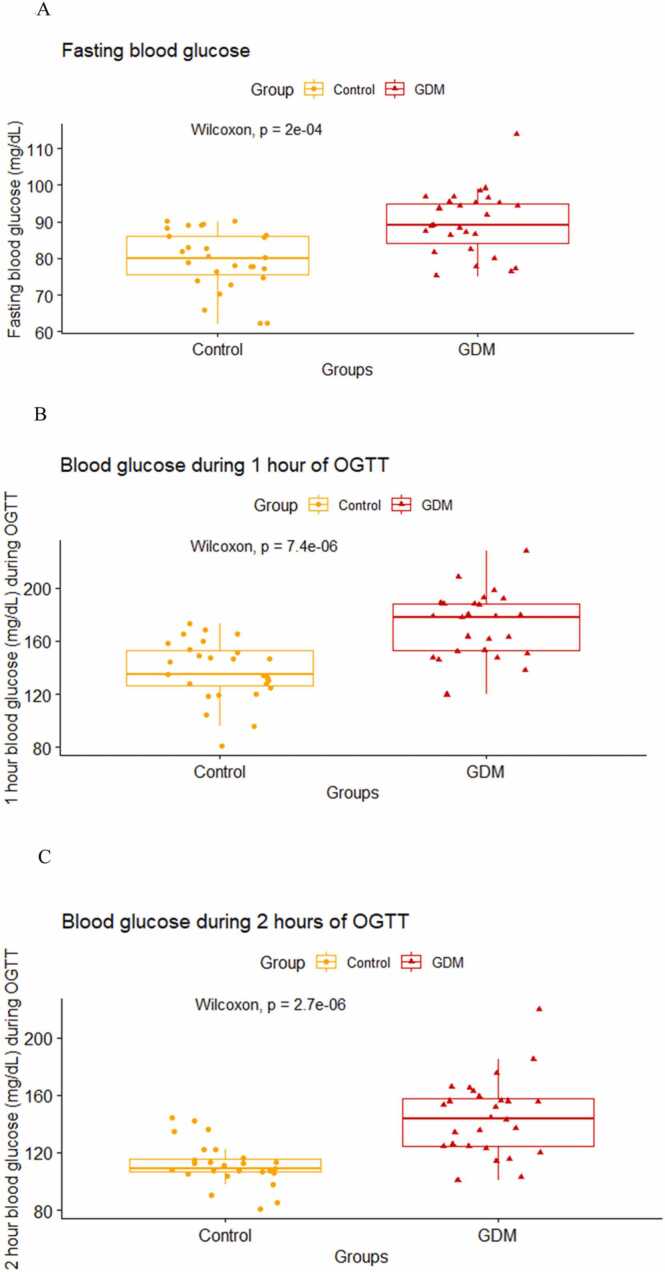
Comparison of median (IQR) blood glucose value during fasting (2 A), at 1 h (2B) and 2 h (2 C) of OGTT between GDM mothers and control using Wilcoxon Rank Sum test (Mann Whitney U test).

All the study participants underwent oral glucose tolerance test during 24–28 weeks of gestation. We found that the fasting blood glucose levels obtained during OGTT were higher in the GDM mothers as compared to the mothers without GDM and it was found to be statistically significant (p < 0.001, Fig. 2A). During OGTT there was a statistically significant difference in the blood glucose levels measured at 1 h (p < 0.001, Fig. 2B) and 2 hours (p < 0.001, Fig. 2C) of post 75 g glucose intake between GDM mothers and mothers without GDM.

## Fasting insulin levels

**Fig. 3:**
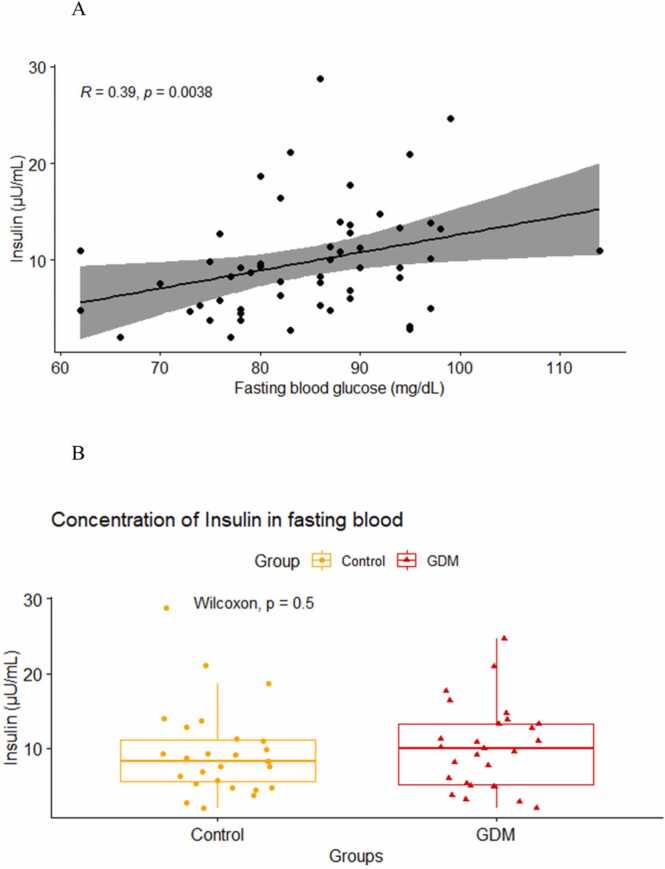
Correlation analysis between insulin and fasting blood glucose (3 A) using Spearman method. Comparison of median (IQR) TNF alpha levels (3B) between GDM mothers and control using Wilcoxon Rank Sum test (Mann Whitney U test).

Serum insulin levels which were known to be elevated in early stages of insulin resistance and type 2 diabetes was measured in both the groups. We found a strong positive correlation between fasting blood glucose levels and fasting insulin levels in pregnant mothers, which was statistically significant (R = 0.39, p < 0.01, Fig. 3A). Fasting insulin levels were slightly higher in GDM mothers [10 (5.1,13.2)] as compared to mothers who didn’t have GDM [8.3 (5.5,11.1)], however, the difference was not statistically significant (p = 0.5, Fig. 3B). HOMA-IR value was found to be slightly higher in GDM mothers [2.10 (1.15, 3.15)] as compared to control mothers [1.70 (1.05, 2.25)] (Table 1). This is indicative of insulin resistance in the GDM mothers; however, the difference was not statistically significant (p = 0.2).

## Biotin concentration in the fasting serum sample

**Fig. 4:**
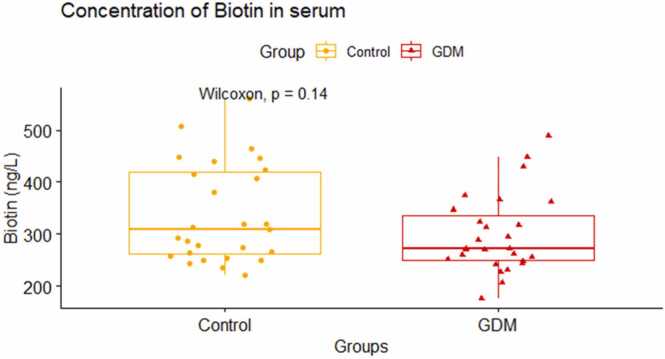
Comparison of median (IQR) biotin levels between GDM mothers and control using Wilcoxon Rank Sum test (Mann Whitney *U* test).

Biotin levels were measured in fasting blood samples of both GDM mothers and mothers without GDM collected during the time of OGTT. We found that the biotin levels were slightly decreased in the GDM mothers [271 (250,335)] as compared to the mothers without GDM [309 (261,419)]. The difference in biotin levels between the two groups was not statistically significant (p = 0.14, Fig. 4). We did correlational analysis for biotin with all the other study variables and found that biotin was not significantly associated with any of the studied variables (Table 2). We did a simple logistic regression analysis to see whether biotin has got any association with the outcome of gestational diabetes mellitus (Table 3). Our analysis showed that biotin levels in the serum has no association with GDM outcome (OR = 0.99, 95 % CI = 0.99–1.00).

**Table 2:** Correlational analysis of biotin with other variables using Spearman method.

Variables	Biotin	
	R	p value
Insulin	-0.045	0.75
Fasting blood glucose	-0.19	0.17
1 hr blood glucose during OGTT	-0.2	0.14
2 hrs blood glucose during OGTT	-0.13	0.33

**Table 3:** Simple logistic regression analysis to see the association of biotin with GDM.

	GDM		
*Predictors*	*Odds Ratios*	*CI*	*p*
(Intercept)	6.07	0.75 – 60.07	0.103
Biotin ng L	0.99	0.99 – 1.00	0.095
Observations	54		
R^2^ Tjur	0.054		

## Discussion

A recent meta-analysis showed that for every one year increase in maternal age from 18, the risk of GDM increases by 7.90 % in the pregnant mothers [18], which is reflected in our study as shown by the increased median age in pregnant mothers with GDM as compared to the pregnant mothers without GDM. We used the one step procedure and IADPSG criteria, which diagnoses GDM when there is violation of any one parameter (fasting, 1 hr or 2hrs blood glucose during OGTT) resulting in high false positivity, however very effective in identifying individuals who are more prone to develop type 2 diabetes in the future [19]. Our results demonstrate that glucose levels measured at all time points such as fasting, 1 h and 2 hrs during OGTT was significantly higher in mothers with GDM as compared to mothers without GDM even though the individual participants rarely violated all three parameters.

Insulin resistance which is defined as decreased biological response to insulin is common in prediabetic state and type 2 diabetes mellitus [20]. Fasting insulin and HOMA-IR which are considered as markers of insulin resistance [21] are slightly elevated in GDM mothers as compared to controls, though it was not statistically significant in our study. Studies have shown that increased HOMA-IR in early pregnancy can be used as a predictive marker of gestational diabetes mellitus and were found to be higher in GDM mothers as compared to control mothers [22], [23].

Biotin levels were found to be lower in individuals with type 2 diabetes and administration of biotin was shown to lower blood glucose level in these individuals without altering the insulin levels [24]. A recent systematic review and meta-analysis on biotin showed that biotin can reduce fasting blood glucose, total cholesterol and triglyceride levels in individuals with type 2 diabetes mellitus [25]. In animal models biotin administration improved the glycaemic status of rats induced with diabetes mellitus [13], [26]. In our previous study we found that mega doses of biotin supplementation improved the pregnancy outcomes in streptozotocin induced gestational diabetes in rats [14]. Biotin levels assessed in serum and urinary excretion of biotin metabolites showed that biotin levels were reduced in pregnant mothers as compared to non-pregnant women [15]. Our study shows that biotin levels were not significantly different between GDM mothers and controls, even though it was slightly higher in control mothers. Our analysis reveals that biotin has no association with blood glucose, insulin, and the outcome of GDM in mothers. Small sample size is the limitation of our study. Including more participants in the study with analysis of biotin levels in different time points in pregnancy may give us directions towards the utility of biotin in gestational diabetes mellitus.

## Funding agency

This study was funded by the Fluid research grant from Christian Medical College, Vellore, India IRB Min No. 11866 obtained by Muthuraman N.

## CRediT authorship contribution statement

PA and MN designed the study. MN collected the sample, did the ELISA assays, analysed, and interpreted the results, wrote the first draft of the manuscript, and revised the edits in the draft. RV recruited the patients, gave critical inputs in the design of the study, edited the manuscript, and approved it. YSP did the biochemical assays, edited the manuscript, and approved it. PC supervised the assays, edited the manuscript, and approved it. PA interpreted the results, helped in drafting the manuscript, edited the manuscript and approved it.

## Conflict of Interest

The authors declare no conflict of interest for this work.
